# Complete mitochondrial genome sequence of the freshwater diatom *Asterionella formosa*

**DOI:** 10.1080/23802359.2017.1285210

**Published:** 2017-02-16

**Authors:** Adrien Villain, Mila Kojadinovic, Carine Puppo, Laura Prioretti, Pierre Hubert, Yizhi Zhang, Gérald Grégori, Alain Roulet, Céline Roques, Jean-Michel Claverie, Brigitte Gontero, Guillaume Blanc

**Affiliations:** a Information Génomique & Structurale UMR 7256, Aix Marseille Univ CNRS, IMM FR 3479, Marseille, France;; b BIP UMR 7281, IMM FR 3479, Aix Marseille Univ CNRS, Marseille Cedex 20, France;; c Laboratoire d'Ingénierie des Systèmes Macromoléculaires, Aix Marseille Univ CNRS UMR 7255 (IMM FR 3479), Marseille, France;; d Mediterranean Institute of Oceanography, Aix Marseille Univ, Univ Toulon, CNRS, Marseille, France;; e GeT-PlaGe, Genotoul, INRA, Castanet-Tolosan, France;; f UAR1209, INRA, Castanet-Tolosan, France;; g Assistance Publique des Hôpitaux de Marseille (APHM), Marseille, France

**Keywords:** Diatoms, Asterionella, mitogenome, pacbio

## Abstract

We report the complete mitochondrial genome sequence of the freshwater diatom *Asterionella formosa*. The large 61.9 kb circular sequence encodes 34 proteins and 25 tRNAs that are universally conserved in other sequenced diatoms. We fully resolved a unique 24 kb region containing highly conserved repeated sequence units, possibly collocating with an origin of replication.

Diatoms are one of the largest and ecologically most significant groups of organisms on the Earth. These unicellular stramenopile algae are broadly distributed in marine and freshwater habitats and studied for potential biotechnological applications as well. *Asterionella formosa* Hassall (Lund 1949) is a freshwater araphid pennate diatom species forming typical star-shaped colonies. A single colony was isolated from Esthwaite Water (54.4N, 2.9W) in the English Lake District in December 2014. DNA was extracted following a hexadecyltrimethylammonium bromide (CTAB)-based protocol, and sequenced using the Pacific Biosciences RSII instrument.

Genome assembly of data from 13 SMRT cells was performed using the HGAP 2.0 protocol (Chin et al. 2013) implemented in SMRT analysis (2.3.0.140936.p0.0). A 82,419-bp long contig was identified as the mitochondrial genome and manually circularized into a 61,877-bp chromosome. Protein-coding genes were predicted by retaining all open reading frames (ORFs) > 100 codons, whereas ORFs <100 codons were only predicted as genes when exhibiting a BLASTP (Altschul 1997) match (*E*-value <1E-5) in the NCBI non-redundant (Nr) database. For all validated genes, start codon predictions were further refined by comparison with homologous sequences. Transfer RNAs were predicted using tRNAscan SE (Lowe & Eddy 1997) and ribosomal RNAs were predicted by alignment with diatom reference sequences. We annotated repeated sequences by combining the results of tandem repeats finder (Benson 1999) and local BLASTN searches. Functional annotations were gathered and manually validated within Unipro UGENE (the UGENE team 2012). The genome sequence is available in DDBJ/EMBL/GenBank under the accession no. KY021079.

The gene content of the *A. formosa* mitogenome is almost identical to previously published diatoms mitochondrial genomes (Secq & Green 2011; Ravin et al. 2010). The 62 genes include small and large rRNAs subunits, 25 tRNAs, and 35 protein-coding genes encoding 16 ribosomal protein subunits (rps), 10 NADH dehydrogenase subunits (nad), 3 ATPase subunits (atp), 3 cytochrome oxidase subunits (cox), the apocytochrome B, and the Sec-independent translocase protein TatC. A single type II intron with an intronic reverse-transcriptase domain is located in the *cox1* gene, while two are found in its homolog in other diatoms. The gene cluster *rps10*-*rps8*-*rpl6*-*rps2*-*rps4*-*atp8*-*rps12*-*rps7*-*rpl14*-*rpl5*-*nad1*-*tatC*-*rps11*-*rpl2*-*rps19*-*rps3*-*rpl16*-*atp9*-*nad4l*-*nad11* is present and seems conserved among diatoms. As in *Phaeodactylum tricornutum*, but unlike *Thalassiosira pseudonana* and *Synedra acus*, most protein-coding genes (32/35 = 91%) are encoded on the same strand. This strong-bias is apparently species-specific as it does not correlate with the phylogenetic relationships shown in Figure 1. Gene density is high and illustrated by overlaps between *rpl2* and *rps19*, and *rps19* and *rps3*. In contrast, a 24.9 kb long region devoid of predicted coding sequences and composed of successive blocks of various tandemly arrayed repeats is located between trnQ and *nad11*.

**Figure 1.**
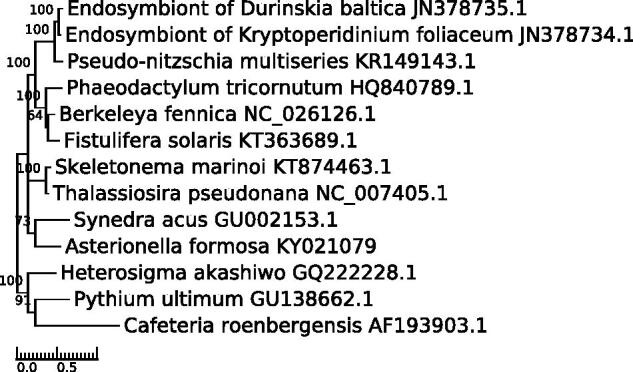
Visualization with TreeGraph (Stöver & Müller 2010) of a phylogenetic tree of selected diatoms and stramenopile mitogenomes based on the concatenation of the protein sequences of the following genes : *atp6*, *atp9*, *cox1*, *cox2*, *cox3*, *nad1*, *nad2*, *nad4*, *nad4l*, *nad5*, *nad6*, *nad7*, *rpl2*, *rps12*, *rps19*, *rps4*, *rps8*. Alignments were performed with MUSCLE (Edgar 2004) and the tree was constructed with PhyML (Guindon et al. 2009) 20120412 using the CpREV model, selected by ProtTest 3 (Darriba et al. 2011). Bootstrap values of 100 permutations are indicated at the nodes.

A single, large repeat region has already been described in other mitogenomes and has been suggested to serve as a replication origin in two cryptophytes (Hauth 2005; Kim et al. 2008). This feature is also typical of diatom mitogenomes (Ravin et al. 2010; Secq & Green 2011), however, neither the sequences nor the general organizations of these repeats are conserved. A few ion torrent-sequenced diatom mitogenomes are reportedly lacking such a repeat region (An et al. 2016a, 2016b) but this absence may be due to the incapacity of short reads to resolve complex repeats, contrary to Sanger or Pacbio sequencing.
